# Investigating the biomechanical function of the plate-type external fixator in the treatment of tibial fractures: a biomechanical study

**DOI:** 10.1186/s12891-020-3144-5

**Published:** 2020-02-27

**Authors:** Di Shi, Kaiyuan Liu, Haomeng Zhang, Xinli Wang, Guochen Li, Lianhe Zheng

**Affiliations:** 1 Department of Orthopedics, the Second Affiliated Hospital of Air Force Medical University, Xi’an, 710038 Shaanxi China; 2 State Key Laboratory for Strength and Vibration of Mechanical Structures, School of Aerospace Engineering, Xi’an Jiaotong University, Xi’an, 710049 Shaanxi China

**Keywords:** Tibial fracture, Plate-type external fixator, Axial compression, Four-point bending, Torsion

## Abstract

**Background:**

The design of an external fixator with the optimal biomechanical function and the lowest profile has been highly pursued, as fracture healing is dependent on the stability and durability of fixation, and a low profile is more desired by patients. The plate-type external fixator, a novel prototype of an external tibial fixation device, is a low profile construct. However, its biomechanical properties remain unclear. The objective of this study was to investigate the stiffness and strength of the plate-type external fixator and the unilateral external fixator. We hypothesized that the plate-type external fixator could provide higher stiffness while retaining sufficient strength.

**Methods:**

Fifty-four cadaver tibias underwent a standardized midshaft osteotomy to create a fracture gap model to simulate a comminuted diaphyseal fracture. All specimens were randomly divided into three groups of eighteen specimens each and stabilized with either a unilateral external fixator or two configurations of the plate-type external fixator. Six specimens of each configuration were tested to determine fixation stiffness in axial compression, four-point bending, and torsion, respectively. Afterwards, dynamic loading until failure was performed in each loading mode to determine the construct strength and failure mode.

**Results:**

The plate-type external fixator provided higher stiffness and strength than the traditional unilateral external fixator. The highest biomechanics were observed for the classical plate-type external fixator, closely followed by the extended plate-type external fixator.

**Conclusions:**

The plate-type external fixator is stiffer and stronger than the traditional unilateral external fixator under axial compression, four-point bending and torsion loading conditions.

## Background

Traditionally, external fixators have been selected as osteosynthesis devices for the treatment of open tibial fractures and certain closed tibial fractures with severe injury to soft tissue [1, 2]. External fixation devices provide a promising and satisfactory alternative for better soft tissue care and for preserving periosteal perfusion to the regions of fracture [3, 4]. They can also be selected for use as interim or definite devices of fracture fixation [4]. However, previous studies have demonstrated that the stiffness of a fixation device is a principal determinant of interfragmentary movement, which has a significant effect on the mechanism and progression of fracture healing [5, 6]. Excessive interfragmentary movement, due to insufficient stiffness of external fixators, can result in deficient callus formation, eventually leading to delayed union or even nonunion with ultimate implant failure [7–9]. Meanwhile, an external fixator with high strength can contribute to durable fixation to allow progressive functional training [10]. In addition, the large profile of the implants, which tends to confer inconvenience to patients during dressing and ambulation, has led to low acceptance of the implants among patients [11, 12].

External fixators used in present clinical practice have various limitations, including insufficient fixation stiffness and strength, leading to poor healing, or high construct profiles, resulting in nonpatient friendly physical burden [13–17]. It is therefore essential to design a novel prototype of an external fixator for tibial fracture to increase rigidity and strength while reducing the profile of the fixation constructs. Our research group designed the plate-type external fixator, a novel prototype of an external tibial fixation device, with a lower profile than traditional unilateral external fixators, which was designed for greater construct stability and durability to treat open tibial fractures and certain closed tibial fractures with severe injury to soft tissue, and we are the first group worldwide to describe such a prototype. In addition, the length of the novel fixator can be adjusted for people of different heights.

Since the biomechanical function of plate-type external fixators remains unclear, this study was performed to investigate the biomechanical parameters of a novel external fixator by comparing with the unilateral external fixator [4, 6, 18]. We hypothesized that the plate-type external fixator would provide higher fixation stability and durability than traditional external tibial fixation devices.

## Methods

### Fracture model

A total of fifty-four fresh and unembalmed tibias were selected from fifty-four voluntarily donated adult male cadavers (Department of Anatomy, Air Force Medical University, Xi’an, China) between the ages of 18 and 50. The average length of the selected tibias was 340 mm (range from 310 mm to 375 mm). All selected tibias specimens were examined for bone mineral density, and osteoporosis was ruled out by means of dual energy X-ray absorptiometry (LUNAR IDAX, GE Inc., Boston, Massachusetts, USA). The tibias were then cleaned of any soft tissues for use in this study. T-scores were selected to represent the values of bone mineral density.

**Fig. 1:**
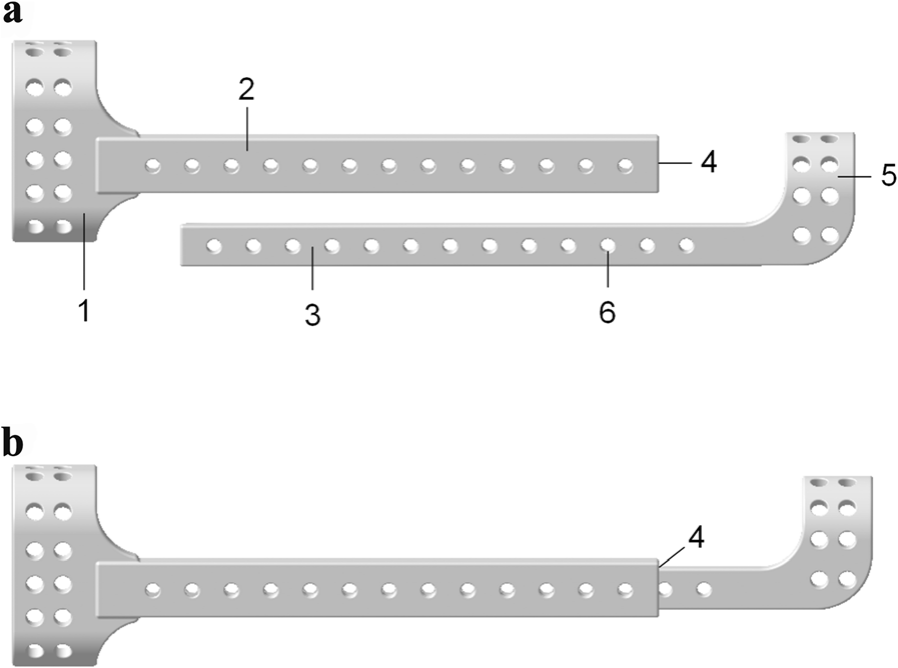
A schematic diagram of the novel external tibial fixation prototype. **a** Partial diagram, **b** global diagram; (1) proximal transverse slat, (2) proximal fixation lath, (3) distal fixation lath, (4) slot, (5) distal transverse slat, and (6) locking screw hole

The novel external tibial fixation device prototype, namely, the plate-type external fixator, consists of a proximal tibial fixation lath with a transverse slat at the proximal end and a distal tibial fixation lath with a transverse slat at the distal end. The distal end of the proximal fixation slat is equipped with a slot, and the proximal end of the distal fixation slat can be inserted into the slot and can slide along lath to adjust the length of the fixator to accommodate various lengths of the human lower limb. In addition, the tibial fixation laths and the transverse slats are both equipped with locking screw holes, and all the screws used are fully threaded self-tapping locking screws (Fig. 1). With a lower profile than the traditional unilateral external fixator, the novel external tibial fixator, designed to match perfectly with the crus, is expected to make it easier to adjust the plate close to the bone surface. For this study, we lengthened the plate-type external fixator by 30 mm, namely, twofold the hole spacing, to clarify whether the extended plate-type external fixator could also provide sufficient stiffness and strength.

The fifty-four tibias were randomly divided into three groups of eighteen specimens each for fixation by a classical plate-type external fixator (CPF), an extended plate-type external fixator (EPF) or the the unilateral external fixator (UEF). Subsequently, the eighteen specimens of each configuration group were randomly divided into three groups of six specimens each for axial compression, four-point bending, and torsion testing, respectively.

A standardized midshaft osteotomy by means of an oscillating saw was performed in all tibias to create a 20 mm fracture gap, measured with the aid of a Vernier caliper, to simulate a comminuted tibial shaft fracture and to ensure no contact between both ends of the fracture. Eighteen specimens were stabilized with a 13-hole stainless steel CPF (300 mm in length, 21 mm in width, 10 mm in thickness, Kangding Medical Alliance Co., Ltd., Shanghai, China), with three 5 mm diameter stainless steel locking screws placed proximally in the first, third and fifth locking holes and three 5 mm diameter stainless steel locking screws placed distally in the ninth, eleventh and thirteenth locking holes. Another eighteen specimens were stabilized with a 15-hole stainless steel extended plate-type external fixator (330 mm in length, 21 mm in width proximally and 16 mm in width distally, 10 mm in thickness proximally and 5 mm in thickness distally, Kangding Medical Alliance Co., Ltd., Shanghai, China), with three 5 mm diameter stainless steel locking screws placed proximally in the second, fourth and sixth locking holes and three 5 mm diameter stainless steel locking screws placed distally in the tenth, twelfth and fourteenth locking holes. Both plate-type external fixators have a hole spacing of 15 mm.

The final eighteen specimens were stabilized with a stainless steel UEF (Kangding Medical Alliance Co., Ltd., Shanghai, China) as the control group. Three stainless steel half-pins (5 mm in diameter) were fixed per fragment and linked with pin clamps to a stainless steel rod (300 mm in length, 11 mm in diameter). The positions of the half-pins corresponded to the proximal locking screws of the CPF in the first, third and fifth holes and to the distal locking screws in the ninth, eleventh and thirteenth holes.

The choice of three locking screws/half-pins per fracture fragment in our study adhered to the AO principles of external fixation that a minimum of three screws were needed to achieve stable fixation on either fragment of the fracture. The AO recommended having a screw near and a screw far from the fracture end in both fragments; however, for the sake of comparison, the most distant screws were inserted into the second and fourteenth locking holes in the extended plate-type external fixator group instead of into the first and fifteenth locking holes, so the same three locking screws/half-pins positions were used in both fragments of the fracture among the three fracture fixation configuration groups. We acknowledge that this represents a limitation of our study, as the adjustment of the locking screws may influence the fixation stiffness of the extended plate-type external fixator.

The offset distance was restricted to 15 mm between the bone surface and the external plates/rods to allow the swelling of soft tissue without disturbance of the configuration and to provide sufficient space for postoperative care. We chose an offset of 15 mm instead of 20 mm or 30 mm for the purpose of increasing the fixation stability of the configuration to prevent excessive interfragmentary movements [4, 19, 20]. The inner locking screws/half-pins were inserted at a distance of 20 mm from the fracture end. The locking screws/half-pins used were long enough to ensure adequate purchase of the bilateral cortex.

### Mechanical testing

**Fig. 2:**
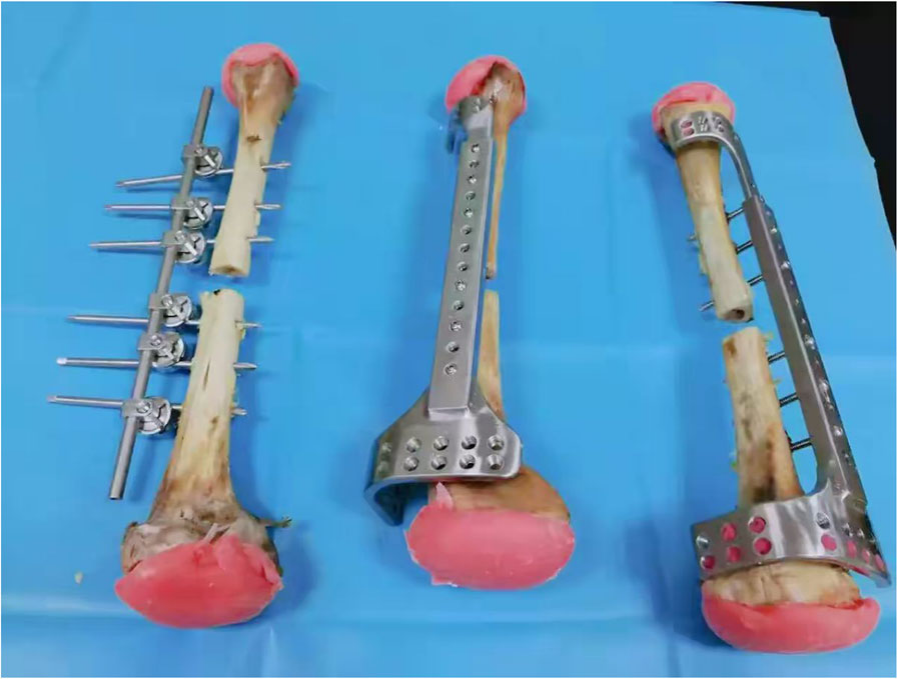
A photograph of the fixation configurations potted in polymethylmethacrylate. Left: unilateral external fixator; middle: classical plate-type external fixator; right: extended plate-type external fixator.

**Fig. 3:**
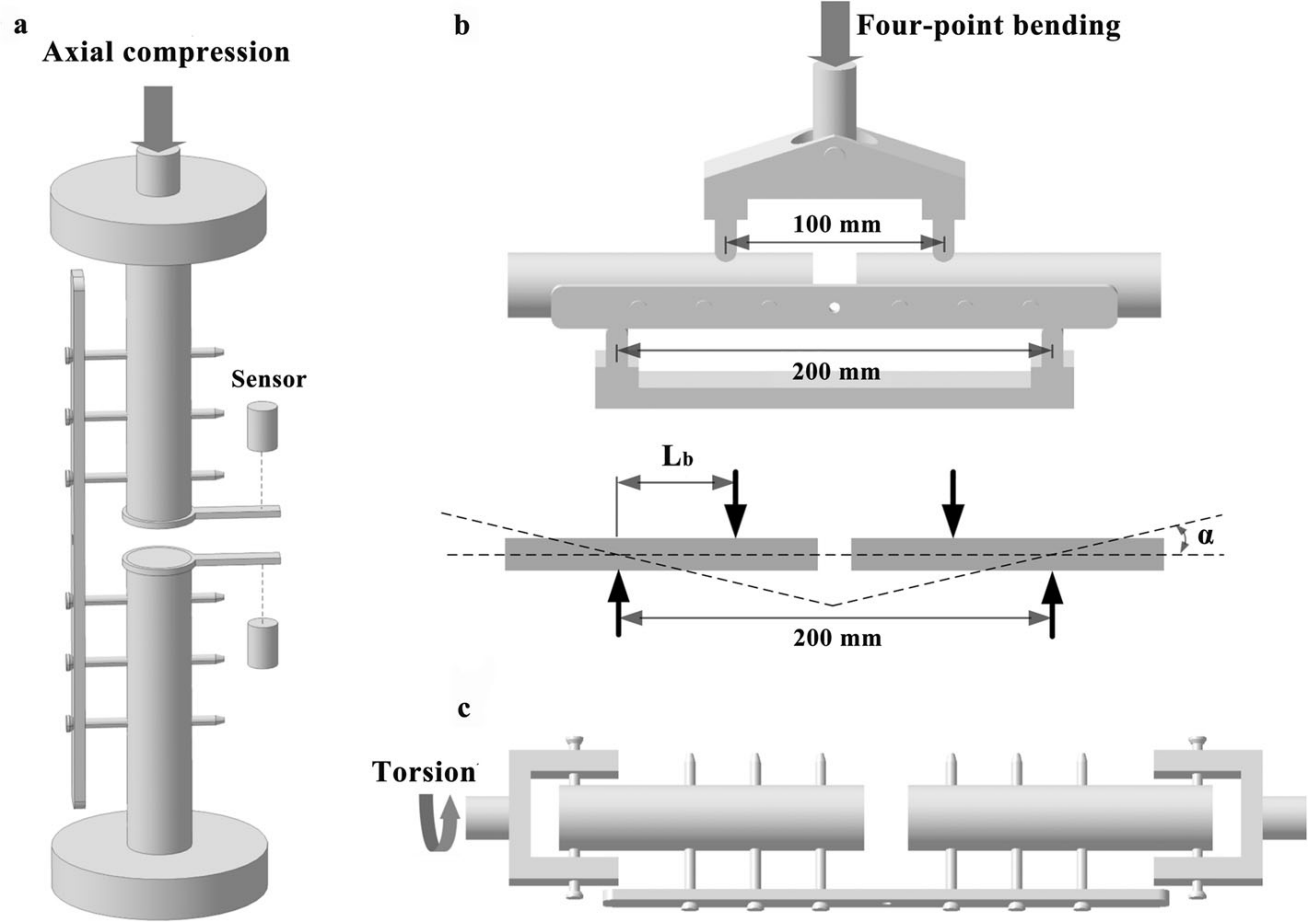
Schematic graphs of the construct stiffness evaluated under three loading conditions. **a** axial compression, **b** four-point bending, and **c** torsion. Lb, bending length; α, bending angle

The proximal and distal ends of all the fracture fixation configurations were potted in polymethylmethacrylate for mechanical testing (Fig. 2) [6]. Subsequently, the bone-implant constructs were mounted in the testing machine with a customized clamp. The classical plate-type external fixation constructs, the extended plate-type external fixation constructs and the unilateral external fixation constructs were tested to determine the fixation stiffness under three loading conditions (axial compression, four-point bending and torsion) (Fig. 3) [6, 21]. The relative displacements at the fracture site were recorded on a computer to calculate the stiffness of the configuration. Subsequently, the three constructs underwent dynamic loading until failure under each loading mode to determine the construct strength and the failure modes. Construct strength was defined as the peak load at the moment of construct failure during progressive dynamic loading to failure under each loading mode. Configuration failure was defined either by catastrophic fracture or by nonrecoverable deformation in the region of fracture, whichever occurred first [5, 22–24].

### Axial compression test

Both ends of the constructs were mounted with the use of a customized axial compression clamp in the Zwick/Roell-Z005 electronic materials testing machine (Ulm, Germany) (Fig. 3a). The applied loading was gradually increased from 0 N to a maximum load of 700 N, corresponding to the weight of a 70 kg person during a one-legged stance [25], at a rate of 0.1 mm/s for six cycles. The interfragmentary displacements at the fracture site were determined by means of laser displacement sensors (LK-G10, KEYENCE Inc., JAPAN). Axial compression stiffness was determined by dividing the axial load values by the vertical displacement values and was expressed in N/mm.

After the static test, sinusoidal loading with a constant load amplitude was applied for each construct. Every 100 loading cycles, the load amplitude was increased stepwise by 100 N until configuration failure occurred, while preloading was applied to 50 N to ensure that construct failure occurred within a reasonable number of loading cycles (< 10,000) by increasing the load stepwise to failure [5, 21].

### Four-point bending test

The constructs were placed in turn by means of a customized bending clamp on a Zwick/Roell-Z005 electronic materials testing machine (Ulm, Germany) (Fig. 3b). The bending moment was calculated by multiplying the bending force by the bending length. The distance between the lower supports was set to 200 mm, while the upper supports were separated by 100 mm. The bending length, defined as the distance between the upper and lower supports on either side of the fracture, was set to 50 mm. The bending force applied was constantly increased up to 400 N, corresponding to a bending moment of 20 Nm, at a rate of 1 mm/min. The bending stiffness was calculated by dividing the bending moment by the bending angle and was expressed in Nm/deg [26, 27]. Afterwards, sinusoidal loading with a constant amplitude was applied for each configuration. The load amplitude was increased gradually every 100 loading cycles by 1 Nm until configuration failure occurred, while the preload was applied to 1 Nm [5].

### Torsion test

The torsional testing was performed by using a CTS-500 microcomputer controlled torsion test machine (Hualong Testing Instrument Co., Ltd., Shanghai, China) equipped with a custom-made torsional clamp, with the proximal and distal ends of the constructs being rigidly clamped by means of the clamp (Fig. 3c). The implemented torque was constantly increased from 0 Nm to 10 Nm at a rate of 0.1 deg/s for six cycles. Torsional stiffness was obtained by dividing the torque value by the relative rotation value and was expressed in Nm/deg. Subsequently, sinusoidal loading with a constant amplitude was applied for each configuration. The load amplitude was increased every 100 loading cycles in steps of 1 Nm until construct failure occurred, while the preload was adjusted to 1 Nm [5].

### Statistical analysis

The collected data were statistically analyzed with SPSS 23.0 software (SPSS, Chicago, Illinois, USA). First, the results were tested for normality and homogeneity of variance. When a normal distribution and homogeneity of variance were found, the data were analyzed by means of one-way analysis of variance to determine the significance of differences in the means among the three groups. The LSD test was used for post hoc testing, if necessary. A *p* < 0.05 was considered statistically significant.

## Results

### Age and bone mineral density

The mean age was similar among the CPF group (33.0 years), the EPF group (31.0 years) and the UEF group (35.5 years). One-way analysis of variance demonstrated that there was no significant difference (*F* = 0.311, *p* = 0.737) among the three fixation groups. The results are displayed in Table 1.

**Table 1:** Mean age and T-score for three fixation configurations

Construct	Number	Age (years)	T-score
		Mean ± SD	Mean ± SD
Classical plate-type external fixator	18	33.0 ± 9.6	−0.81 ± 0.09
Extended plate-type external fixator	18	31.0 ± 10.9	− 0.84 ± 0.10
Unilateral external fixator	18	35.5 ± 9.2	−0.83 ± 0.09

There was also no significant difference (*F* = 0.100, *p* = 0.905) in the mean T-score among the CPF group (− 0.81), the EPF group (− 0.84), and the UEF group (− 0.83). Table 1 displays the results. Since the mean T-score values of the three fixation groups were all greater than − 1, we can conclude that all the specimens were normal bone and excluded of osteoporosis.

### Construct stiffness

**Fig. 4:**
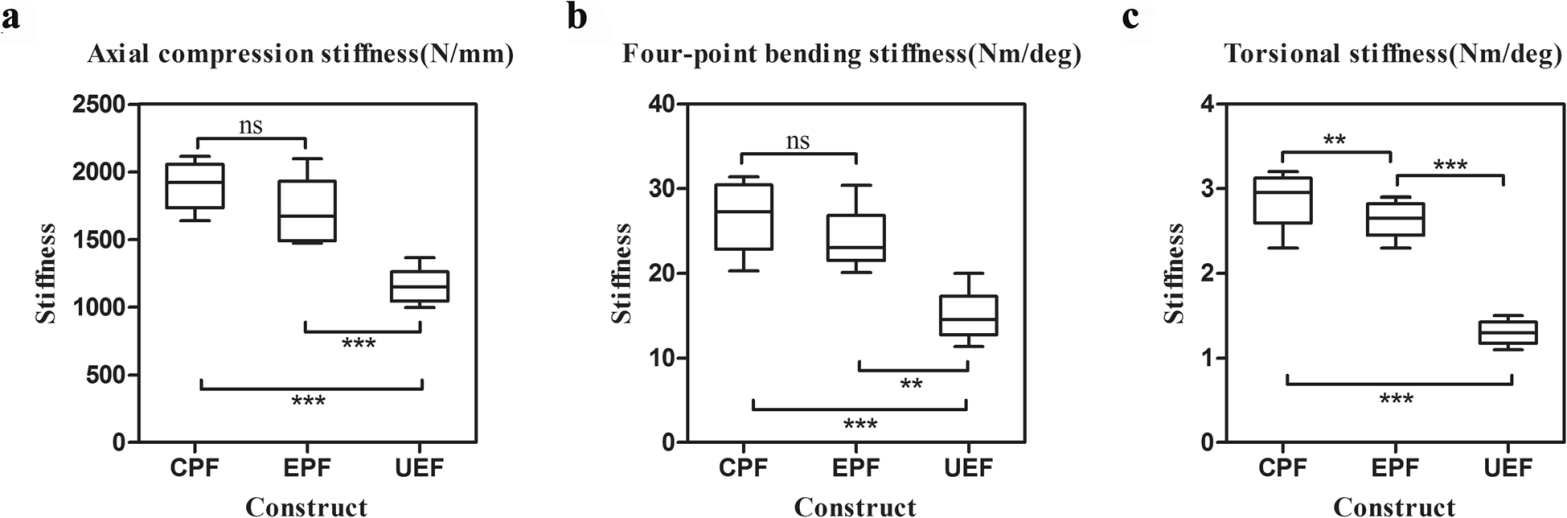
Box plots of the three fixation configurations under axial compression (**a**), four-point bending (**b**) and torsion (**c**). CPF, classical plate-type external fixator; EPF, extended plate-type external fixator; UEF, unilateral external fixator. ** indicates statistical significance (*p* < 0.01); *** indicates statistical significance (*p* < 0.001); ns indicates no significant difference

One-way analysis of variance demonstrated that the mean axial stiffness was significantly (*F* = 24.642, *p* < 0.0001) different among the CPF group (1898.8 N/mm), the EPF group (1715.8 N/mm), and the UEF group (1157.8 N/mm). The axial stiffness parameters of the three groups are displayed in the chart in Fig. 4a. The LSD test revealed that the axial stiffness of the CPF group was significantly (*p* < 0.0001) higher than that of the UEF group by 0.64. The axial parameter of the EPF group was also significantly (*p* < 0.0001) higher than that of the UEF group by 0.48.

There was a significant (*F* = 17.365, *p* < 0.0001) difference in the mean four-point bending stiffness among the CPF group (26.7 Nm/deg), the EPF group (24.1 Nm/deg), and the UEF group (15.0 Nm/deg). The chart in Fig. 4b shows these values. The post hoc LSD tests were performed for pairwise comparisons, which revealed that the bending stiffness of the CPF group was significantly (*p* < 0.0001) greater than that of the UEF group by 78%. The stiffness value of the EPF group was also significantly (*p* = 0.001) greater than that of the UEF group by 61%.

One-way ANOVA revealed significant (*F* = 130.824, *p* < 0.0001) differences in mean torsional stiffness among the CPF group (3.0 Nm/deg), the EPF group (2.6 Nm/deg), and the UEF group (1.3 Nm/deg). The results are displayed in Fig. 4c. The subsequent LSD test demonstrated that the torsional stiffness of the CPF group was significantly (*p* < 0.0001) greater than that of the UEF group by 1.31. The stiffness value of the EPF group was also significantly (*p* < 0.0001) greater than that of the UEF group by 1.

### Construct strength

**Fig. 5:**
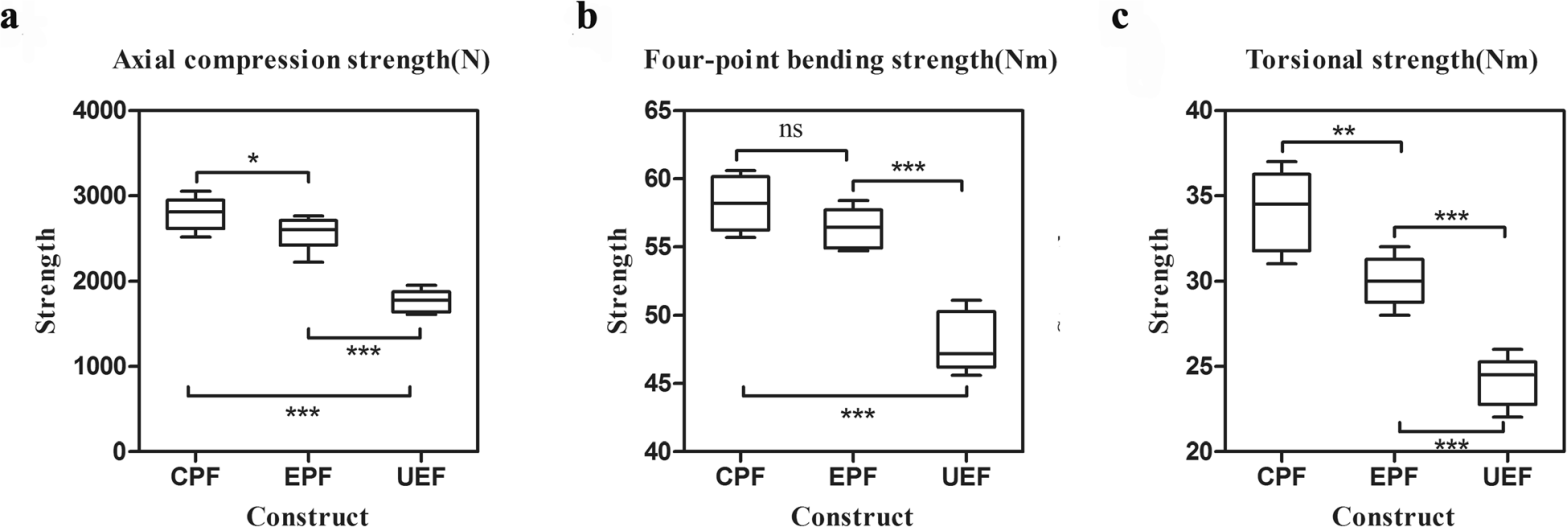
Box plots of the three fixation configurations under axial compression (**a**), four-point bending (**b**) and torsion (**c**). CPF, classical plate-type external fixator; EPF, extended plate-type external fixator; UEF, unilateral external fixator. * indicates statistical significance (*p* < 0.05); ** indicates statistical significance (*p* < 0.01); *** indicates statistical significance (*p* < 0.001); ns indicates no significant difference

**Fig. 6:**
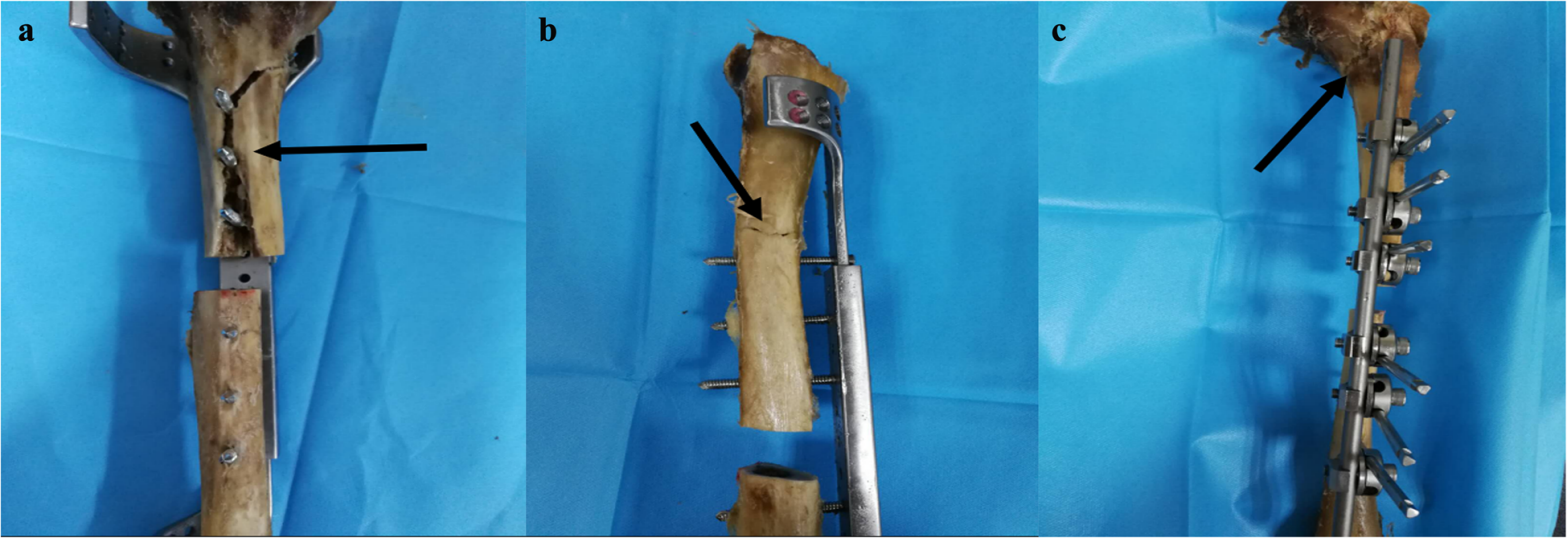
Photographs of the failure modes of configurations for **a** axial compression, catastrophic fracture of the diaphysis through the screw hole found in the classical plate-type external fixator group (indicated by the black arrow); **b** four-point bending, catastrophic fracture of the diaphysis found in the extended plate-type external fixator group (indicated by the black arrow); and **c** torsion, oblique fracture found in the unilateral external fixator group (indicated by the black arrow)

For axial compression, the strength of the CPF group (2792.2 N) was significantly (*p* < 0.0001) higher than that of the UEF group (1769.0 N) by 0.58. The axial parameter of the EPF group (2560.5 N) was also significantly (*p* < 0.0001) greater than that of the UEF group by 0.45. The results are displayed in Fig. 5a. Both CPF and EPF constructs failed by catastrophic fracture of the diaphysis through the screw hole (Fig. 6a). After fracture, the CPF constructs in four specimens displayed no implant hardware failure, screw breakage occurred in one specimen, and screw bending occurred in one specimen. Among the EPF constructs, no implant hardware failure was found in one specimen, screw and plate bending were observed in three specimens, and screw breakage occurred in two specimens. The UEF constructs failed as a result of nonrecoverable fracture gap closure due to half-pin and rod bending in five specimens and due to fracture of the diaphysis in one specimen.

In four-point bending, the construct strength of the CPF group (58.2 Nm) was significantly (*p* < 0.0001) greater than that of the UEF group (47.9 Nm) by 22%. The strength value of the EPF group (56.4 Nm) was also significantly (*p* < 0.0001) higher than that of the UEF group by 18%. A chart of these results is shown in Fig. 5b. All constructs failed by catastrophic fracture of the diaphysis (Fig. 6b). After fracture, none of the CPF constructs displayed implant hardware failure; the EPF constructs showed no implant hardware failure in three specimens and screw bending in three specimens; and the UEF constructs showed half-pin and rod bending in four specimens and half-pin breakage and rod bending fracture in two specimens.

For torsion, the strength of the CPF group (34.2 Nm) was significantly (*p* < 0.0001) greater than that of the UEF group (24.2 Nm) by 0.41. The strength value of the EPF group (30.0 Nm) was also significantly (*p* < 0.0001) greater than that of the UEF group by 0.24. The results are displayed in Fig. 5c. The CPF constructs failed by screw and plate bending in four specimens, screw breakage in one specimen and spiral fracture in one specimen. The EPF constructs failed as a result of screw and plate bending in two specimens, screw breakage and plate bending in two specimens and spiral fracture in two specimens. The UEF constructs exhibited oblique fracture in four specimens (Fig. 6c) and half-pin and rod bending in two specimens, resulting in nonrecoverable deformation in the region of fracture.

## Discussion

The results of this study support the hypothesis that the plate-type external fixator can remarkably increase the stiffness of the fracture fixation construct while retaining sufficient strength. In this experiment, the two configurations employing the plate-type external fixator exhibited higher stiffness and strength than the traditional UEF in axial compression, four-point bending and torsion. Furthermore, our study showed that the stiffness and strength of the construct would decrease with thinner plate thicknesses. However, the extended plate-type external fixator was still stiffer and stronger than that of the traditional UEF.

### Construct stiffness

The stiffness of external fixators reported in previous literatures has ranged from 50 N/mm to 2500 N/mm in axial compression, 10 Nm/deg to 100 Nm/deg in four-point bending, and 1 Nm/deg to 4 Nm/deg in torsion, respectively [4, 6, 28–31]. The stiffness values of all of the bone-implant constructs in our study research were within these ranges, and the plate-type external fixator provided remarkably higher torsional stiffness than the UEF. The highest stiffness was achieved in the fracture model with an offset distance of 5 mm, while the lowest stiffness was achieved with distances up to 30 mm. Our study reveals the possibility that stiffness may decrease with increasing offset.

### Construct strength and failure mode

According to our study, we conclude that the plate-type external fixator is stronger than the UEF, thus contributing to more durable fixation to allow progressive functional training of greater intensity and duration. Moreover, for progressive dynamic loading to failure, the UEF group displayed the greatest implant hardware failure and the minimum peak load.

### Factors infecting the stiffness and strength

Previous studies that have investigated how fixation stiffness and strength can be influenced have shown that several factors affect the stability and durability of bone-implant constructs [19, 29, 32–37]. The working length, defined as the distance between the first two screws on both sides of the fracture gap, has a marked impact on biomechanical fixation. Meanwhile, altering the offset distance between the plate and the bone surface can significantly change the stability and durability of the bone-implant construct. Moreover, the number and positions of the screws, the fracture gap size, and the material properties of the external fixator all influence the biomechanical parameters. In our study, the working length of the three fixation groups was set to 60 mm, namely, fourfold the hole spacing. A 15 mm offset was maintained between the plate/rod and the bone surface in the three fixation groups. In addition, the number of screws, the fracture gap size, and the material properties were the same in the three external fixation groups. Therefore, it can be concluded that the plate-type external fixator provides more sufficient stability and durability than the UEF because the stiffness and strength of the plate-type external fixator were higher in axial compression, four-point bending and torsion.

### Implications of the novel fixator

The plate-type external fixator described herein by our research group is the first such prototype to be described worldwide. These tests represent the first biomechanical study comparing the stiffness and strength of the novel fixator with the UEF. In addition to the higher stiffness and strength and the lower profile, the plate-type external fixator, which is designed to match perfectly with the crus, provides sufficient skin distance by allowing the distance between the bone surface and the external plate to be adjusted. In addition, the length of the novel fixator can be adjusted for people of different heights. When applying the novel fixator to treat tibial fractures without an open approach, we need to make closed reduction a priority; if unsuccessful, limited exposure is needed.

### The angle-stabilizing property

The locking plate, which is based on an angle-stabilizing property, is used as an external fixator and can be considered as a promising and satisfactory procedure, which has already been reported in many literatures [1, 2, 38, 39]. The plate-type external fixator, also depending on the angle-stabilizing property, is expected to yield excellent clinical results when used for treating tibia fracture.

### Limitations

There remain several limitations of this study. First, the investigation of stiffness and strength was performed in vitro, and all specimens were cleaned of any soft tissues; therefore, in the load applied in this fixation model may not completely stimulate the multifaceted load pattern in vivo. Second, the fixation parameters were only investigated for nonosteoporotic specimens. Ideally, we the biomechanical parameters for osteoporotic specimens should also be investigated, since stiffness and strength are highly affected by bone quality.

### Feasibility and practicality

Despite the aforementioned limitations, we believe that the model used in this study is appropriate for comparing stiffness and strength between the plate-type external fixator and the UEF. The biomechanical parameters were investigated individually for the main load bearings that a fracture-fixator configuration might sustain, namely, axial compression loading, bending loading and torsional loading. These parameters are extremely useful for developing a comprehensive understanding of the relative benefits of plate-type external constructs under in vivo multifaceted loading modes, which comprise some of the combinations of forces investigated herein. Therefore, we believe that the results of this study are appropriate for extrapolation to human applications and can be applied to make clinical judgments.

## Conclusions

The design of an external fixator with optimal biomechanical function and a low profile has been a research priority. In this study, we found that the was significantly stiffer and stronger than the traditional UEF, and the stiffness of plate-type external fixator was closer to the optimal value. Moreover, the low profile of the plate-type external fixator reduces inconvenience during dressing and ambulation, thus increasing comfort and improving its acceptance among patients. In conclusion, the plate-type external fixator provides a promising and satisfactory alternative to the traditional UEF, since its sufficient stiffness and strength and lower profile.

## Data Availability

All data supporting the results of this study are available within the article.

## Abbreviations

ANOVA: Analysis of variance
CPF: Classical plate-type external fixator
EPF: Extended plate-type external fixator
UEF: Unilateral external fixator
